# The interaction of socioeconomic status with place of death: a qualitative analysis of physician experiences

**DOI:** 10.1186/s12904-018-0341-1

**Published:** 2018-06-20

**Authors:** Joshua Wales, Allison M. Kurahashi, Amna Husain

**Affiliations:** The Temmy Latner Centre for Palliative Care, Sinai Health System, 60 Murray Street, 4th Floor, Box 13, Toronto, ON M5T 3L9 Canada

**Keywords:** Palliative care, Social class, House calls, Socioeconomic factors, Healthcare disparities, Place of death

## Abstract

**Background:**

Home is a preferred place of death for many people; however, access to a home death may not be equitable. The impact of socioeconomic status on one’s ability to die at home has been documented, yet there remains little literature exploring mechanisms that contribute to this disparity. By exploring the experiences and insights of physicians who provide end-of-life care in the home, this study aims to identify the factors perceived to influence patients’ likelihood of home death and describe the mechanisms by which they interact with socioeconomic status.

**Methods:**

In this exploratory qualitative study, we conducted interviews with 9 physicians who provide home-based care at a specialized palliative care centre. Participants were asked about their experiences caring for patients at the end of life, focusing on factors believed to impact likelihood of home death with an emphasis on socioeconomic status, and opportunities for intervention. We relied on participants’ perceptions of SES, rather than objective measures. We used an inductive content analysis to identify and describe factors that physicians perceive to influence a patient’s likelihood of dying at home.

**Results:**

Factors identified by physicians were organized into three categories: patient characteristics, physical environment and support network. Patient preference for home death was seen as a necessary factor. If this was established, participants suggested that having a strong support network to supplement professional care was critical to achieving home death. Finally, safe and sustainable housing were also felt to improve likelihood of home death. Higher SES was perceived to increase the likelihood of a desired home death by affording access to more resources within each of the categories. This included better health and health care understanding, a higher capacity for advocacy, a more stable home environment, and more caregiver support.

**Conclusions:**

SES was not perceived to be an isolated factor impacting likelihood of home death, but rather a means to address shortfalls in the three identified categories. Identifying the factors that influence ability is the first step in ensuring home death is accessible to all patients who desire it, regardless of socioeconomic status.

## Background

Home has emerged as the preferred place of death for most patients [1–3] and as a cost-effective alternative to institutionalized death [4]. Studies have shown that access to home death is influenced by a number of personal factors, including age [5, 6], sex [5], education [7], disease type [8, 9], marital status or cohabitation [6, 10–12], preferred place of death [13], ethnicity [8, 10], informal support networks [6, 14], and cultural affiliations [8]. Access to care in the home has also been associated with home death. Previous studies investigating the availability and utilization of home care and home visits found that patients were more likely to die at home or out of acute care if they lived in an area with more home hospice providers [10], received home care in the last 6 months of life [15], or received more nursing and personal support worker visits [6, 16, 17].

Associations between socioeconomic status (SES) and place of death in the literature are inconsistent. Several studies found that rates of home death are lower for patients defined as lower SES or low income [10, 12, 17–24]. Conversely, other studies did not find a significant association between place of death and socioeconomic status based on perceived or actual income level [11, 25–29], deprivation score [30], or education level [31].

Previous studies that have identified SES-dependent differences in rates of home death have hypothesized factors to explain these disparities: patient or family capacity to provide adequate care at home [18, 22], level of access to care services [20, 22], personal preferences [22], care-related costs [18], control over monetary resources [23], and ability to secure local supports [20]. Few studies, however, have directly explored the mechanisms by which these determinants – including SES – result in disparities of home death rates. A more complete understanding of these socioeconomic determinants of home death may provide some insight into the inconsistent findings in the literature and help to address SES-related inequities that may exist in accessing home death.

This exploratory study aims to identify the key factors perceived to influence likelihood of home death and define the mechanisms by which they interact with SES. The findings presented here are based on the experiences of physicians who provide home-based palliative care in an urban Canadian context. In future research, we will use the factors identified in this study to refine the research question and capture the perspectives of patients, families and other healthcare providers. They may also highlight areas of opportunity for policy and practice development to mitigate potential disparities within a home-based palliative care context.

## Methods

### Study design

Working within a Qualitative Description framework [32], the investigators used inductive qualitative content analysis [33] to describe the factors that may influence the likelihood of home death and describe how these factors interact with patient socioeconomic status. Qualitative description studies aim to provide a comprehensive summary of a phenomenon that can be used as an entry point for further study [32]. Our data were informed by physician’s perceptions of their patients’ SES and did not employ empiric indices of patients’ socioeconomic statuses. At the time of recruitment, the principal investigator (JW) was completing his residency and was known to participants as a colleague and student. He currently practices as a staff physician at the centre. The study coordinator (AK) has experience conducting research interviews but is not a clinician. She was known by all participants. The study protocol was approved by the local Research Ethics Board. The methods and findings are described according to the consolidated criteria for reporting qualitative research (COREQ) guidelines [34].

### Population

We interviewed physicians whose clinical practice is focused on providing palliative care services to individuals at home. Physicians providing home-based palliative care may be well placed to comment on the determinants of home death. First, they can compare between their experiences and interactions with different patients and patient support networks, which occur in a home environment. Second, their experiences leading goals of care discussions can inform insights into the decision-making processes around place of death. Finally, they can root their perceptions about patient SES in their experiences discussing financial capacity with patients when planning care services.

In order to generate a deep understanding of the factors perceived within a home-based palliative care context, we recruited physicians from a single palliative care centre in Toronto, Ontario. The centre is one of the largest home palliative care programs in Canada, and offers 24/7 physician support to patients with life-limiting illness in their homes. Seventeen physicians dedicate an average of 13 full time equivalents (FTEs) to home care practice. Physicians have had a focused practice in palliative care at the centre ranging from 1.5 to 20 years. The area served by the palliative care centre has nearly equal distribution between income quartiles [16] but incudes neighbourhood clusters of both low- and high-income households [35]. The catchment area is divided into smaller zones, which are serviced by one or two physicians each. Because each zone has a distinct socioeconomic and cultural make-up, some physicians may service areas with a higher density of low-income households than other physicians.

In 2016, the centre recorded 12,551 encounters (physician calls or home visits) with patients. Both types of encounters are necessary to provide palliative care in patients’ homes. On average, there were 80.5 encounters per physician FTE per month, and each patient experienced an average of 5.9 encounters.

### Sample size and recruitment

The principal investigator sent an email invitation to participate in the study to all physicians practicing with the palliative care centre who provide home-visits. Interested physicians contacted the study coordinator to schedule an interview. Of the 17 physicians contacted, 9 participated in the study. Four physicians were not responsive, three had scheduling conflicts, and one declined as they felt their experiences would not contribute to understanding the research question. Participants were made aware of JW’s role in the study and the safeguards that were used to preserve privacy during the consenting process.

### Data collection

The semi-structured interview guide was created and refined by JW and AK through a process of evaluating each question’s relevance to the overall study aim. The final interview guide (Appendix 1) consisted of a mix of open ended and quantitative questions. An interview preamble informed patients that the researchers were interested in the experiences of home-care palliative physicians and the factors they believe to influence a patient’s ability to die at home. Participants were not given definitions for the terms “high” and “low” socioeconomic status, nor patient economic data. Earlier questions probed physicians’ general experiences and factors believed to influence the likelihood of home death. Later questions targeted factors related to socioeconomic status, discrepancies resulting from SES, and strategies to address these discrepancies. Individual or telephone interviews were conducted during February 2016. AK conducted all interviews in order to protect participant confidentiality and reduce potential response bias that may have presented if the principal investigator had conducted the interviews.

### Data analysis

All interviews were audio recorded, professionally transcribed verbatim and de-identified to maintain participant privacy. AK compared completed transcripts to audio recordings to verify accuracy. Based on AK’s field notes, the researchers periodically met to discuss emerging concepts, noting ones that were new or redundant with earlier interviews. Based on these reviews, greater focus was placed on questions probing socioeconomic status and caregiver support levels after the first 6 interviews were completed. Three more interviews were conducted, but no new concepts emerged. Two reviewers (JW & AK) independently open-coded the first three interview transcripts using NVivo version 10 software, generating detailed, non-hierarchical lists of codes. The reviewers then met to compare and refine their list of open codes (64 codes). At this time, duplicate codes were merged and the resulting 37 codes were defined and organized into five categories: Patient Factors, Caregiver Factors, System Factors, Socioeconomic Factors and Strategies to decrease disparity. The reviewers independently coded all nine interview transcripts according to this coding framework, applying all relevant codes to capture interactions between factors. The reviewers met again to compare their application of codes. Any discrepancies were resolved through discussion. Data within each category were reviewed and grouped into sub-categories [33], which represent key factors. The researchers used visual schemata to conceptualize the relationships between factors within and between categories. Factors were continually compared against the primary data to ensure accurate representation. This iterative process of categorization and comparison was continued until no new factors emerged. Because the consistency of concepts across interviews demonstrated the stability of the coding framework (i.e. no new codes were added), and because no new factors were emerging from ongoing data abstraction, the researchers agreed that saturation had been reached [33, 36–38], and that no additional interviews were required. If, following analysis, saturation had not been reached, efforts would have been made to schedule interviews with those physicians who previously had scheduling conflicts. Given JW’s position at the palliative care centre, efforts were made to recognize his own personal clinical experiences, and how they informed the analysis of the data. Any conclusions drawn were discussed with the study coordinator and examined against the data to ensure they were not unduly coloured by biases derived from his clinical experience.

Counts and percentages were calculated for Yes/No questions. Median and range were calculated for the question asking about percentage of patient population felt to be of lower SES.

## Results

Participant responses to quantitative questions are presented in Table 1. All participants indicated that they had cared for patients they would consider to be of lower SES. When describing their current practice, participants indicated that on average, approximately 30% of their patients (range 7.5–90%) could be considered to be of lower socioeconomic status, reflecting the different areas served by each physician. All but one participant (89%) indicated that they believed socioeconomic status had an impact on a patient’s ability to die at home.

**Table 1:** Participant quantitative responses

Participant	Q5: Have you ever cared for patients you would consider to be of lower socioeconomic status? (Yes or No)	Q6: What percentage of your current patent population would you consider to be of lower socioeconomic status? (%)	Q7: Do you feel that there is a difference in a patient’s ability to die at home depending on if they are of lower or higher socioeconomic status? (Yes or No)
1	Yes	30	Yes
2	Yes	38	Yes
3	Yes	33	Yes
4	Yes	5–10	Yes
5	Yes	90	Yes
6	Yes	10–20	No
7	Yes	90	Yes
8	Yes	20	Yes
9	Yes	20–25	Yes

During the 20–40 min interviews, physicians described several factors that they perceived to impact ability to die at home. These factors were grouped into three categories: patient characteristics, physical environment, and support network (Table 2). While a higher perceived SES was not identified as a primary determinant of a successful home death, it was consistently noted to strengthen the other key categories that were identified. Below, we describe each factor, how they are perceived to interact with SES, and possible strategies to decrease disparities in accessing home death.

**Table 2:** Identified categories and factors

Category	Definition	Factors
Patient Characteristics	Characteristics related to a patient’s background, culture and character traits, as well as their physical, emotional or mental state were believed to influence their ability to die at home. These were described by 8/9 participants.	1. Patient preferences about place of death 2. Patient ability to navigate the health care system
Physical Environment	A patient’s physical environment refers to the location (home or housing) where they will receive palliative care. Characteristics pertaining to the physical environment were believed to influence likelihood of dying at home. This was described by 8/9 participants.	1. Environment suitability to accommodate care 2. Environment stability 3. Environment safety
Support Network	The support network includes any individual who provided care or assistance to the patient during their end-of-life care trajectory. Having more support was equated with a greater likelihood of dying at home. This was described by 9/9 participants.	1. Caregiver availability and ability to organize and coordinate services 2. Ability to supplement care needs with paid-caregiving services 3. Health care provider advocacy for patient needs

### Patient characteristics

Two patient characteristics were believed to influence a patient’s ability to die at home: their preferences about place of death, and their ability to navigate the health care system. These factors were informed by perceptions of a patient’s background, culture and character traits as well as their physical, emotional or mental state that were felt to impact their likelihood of dying at home.

Patient preference was seen as a key determinant of place of death. Physicians reported that patient preference for home death was often determined by emotional factors, such as patient and caregiver discomfort, fear, or anxiety. These emotions were mainly centred around fear of coping poorly, perceived or actual burden on caregivers, and the potential lasting impact of home death on family members.

“There are some people for whom it’s not a number of hours or amount of money issue, they just don’t feel comfortable dying at home”… “When given the choice of palliative care unit or home, and somebody doesn’t want to put their family through something, it doesn’t matter if they can hire caregivers or not... because it’s the emotional aspect of dying at home. Sometimes I’ve been told, you know, if I die here so-and-so won’t be able to sleep here anymore.” (Participant 1)

Many respondents attributed patient fear about home death to poor illness understanding, which was associated with perceived lower levels of education, language barriers or lack of access to resources.

“If [patients] don’t really understand or know what to expect, then they’re easily anxious and thus call a lot or go to hospital a lot because they just don’t feel comfortable being at home, even though, probably, some aspects of that care can be done quite adequately at home. They just can’t deal with that.” (Participant 5)

Beyond preference, participants noted that patients required the ability to navigate the health care system, advocate for their health care needs, and assemble a support network. Participants felt that ability to advocate was influenced by a patient’s level of education, socioeconomic status, and understanding of their disease trajectory.

“I think the other factor, too, is whether they have somebody advocating for them, so whether they are able to know who to talk to and how to access care... Those who may not have English as the first language or those who are new, don’t know who to turn to or who to ask… They don’t know if they should be asking or can ask.” (Participant 3)

### Physical environment

Factors relating to a patient’s physical environment primarily pertained to the suitability, stability and safety of their housing for meeting the needs of patients and caregivers at the end of life. A physical environment was perceived to be suitable to support home death if it was able to accommodate medical equipment and care providers, had access to the basic amenities (e.g. toilet, shower), and was free of hazards (e.g. bedbugs, vermin, mould).

“Sometimes a place is so small or cluttered that you can’t put a hospital bed in, or sometimes there are vermin in the dwelling, so [also] physical factors of them not being able to get from one place to the other, not having access to the washroom or a shower.” (Participant 1)

Participants felt that patients living without access to reliable or subsidized housing were less likely to die out of an institutionalized setting.

“It’s not a widespread problem but for the people who don’t have access to housing, it is a big problem” (Participant 9)

Socioeconomic status was perceived to impact these factors, insofar as poverty was associated with less suitable and more precarious housing.

The safety of the home environment also influenced the ability or willingness of providers to care for patients at home. The safety concerns described were typically associated with lower-income situations, and included residing in neighbourhoods with run down buildings, a higher prevalence of mental health issues, and substance abuse issues.

“The other piece is safety. In a lot of these places, can we have things like opioids and controlled substances in a place where if they’re alone they need to keep the door unlocked and so who is going to go in and potentially affect them… And then not only myself as a physician but the nursing staff, the safety and a lot of these places unfortunately are deemed unsafe.” (Participant 3)

These perceived and actual concerns for personal safety may preclude providers from participating in a patient’s after hours care.

“One of the barriers, especially in my area is a lot of those areas are what we call ‘no go’ zones at night. So, if someone calls after hours or when it’s dark, a doctor is not going to go out to see them because it’s unsafe.” (Participant 5)

Some physicians acknowledged that what constituted a suitable environment for home death can be subjective. When describing patients living in less-suitable environments, some participants appeared to remain objective when evaluating whether a patient might be able to die there.

“In some cases you don’t impose your sense of what a good home is, and I may not be willing to live in that kind of environment.... You can’t impose-- That’s not how I would want to live or want to care for someone. I guess we kind of have insight about what we think we might need, so the running water and the shower and room to turn and manoeuvre and all the things that we think the person might need as they get sicker.” (Participant 6)

Other physicians suggested that care provider bias about a patient’s environment might impact where that patient ultimately dies.

“The sad thing is that it’s so integrated into how I think about patients now, almost right from the get go. I will have a sense of ‘is this going to work or is it not’. And it’s not like I would say it’s absolutely not going to work if the patient has very limited finances, but it’s something that we address much more quickly in the visits. I almost wonder: do we end up steering the patient away from an anticipated home death because we can tell [that it will be difficult to provide end of life care].” (Participant 2)

### Support networks

Support networks included any individuals who provided care or assistance to the patient during their end-of-life care trajectory. Participants highlighted three support-related factors that improved a patient’s likelihood of dying at home: having family caregivers who are available and able to organize and coordinate services, being able to supplement care needs with paid-caregiving services, and having health care providers who would advocate for patient needs.

“I would say if the family and the patient, but mainly the family, are committed to making it happen at home, and they have enough numbers, possibly more than one committed family member to share the load, then I think it’s possible.” (Participant 2)

Like the physician above, all respondents highlighted the presence of a caregiver as the most significant factor in achieving a home death, if that is what the patient desires. Physicians noted the key role of caregivers as advocates who organized and coordinated service delivery. Socioeconomic status was perceived to influence caregiver support in two ways. Frist, participants believed that family members who are unable to take time off work for economic reasons are less available to be primary caregivers.

“I think one of the real kind of hidden ways in which socioeconomics comes into play, is when caregivers need to work in order to keep the food on the table and the rent and the hydro paid and all that… So I would love to see a situation where we supported caregivers financially to do caregiving.” (Participant 9)

Second, participants felt that caregivers’ abilities to advocate were associated with education level, language skills, and familiarity with the health care system. Caregiver emotional factors were also significant; discomfort with home death, or inability to cope with the demands placed on caregivers were frequently cited reasons for transitioning to an institutional setting.

Patients that were perceived to have more financial resources were frequently able to compensate for a lack of informal caregiver support by purchasing private care. This ability to afford private services was consistently described as the primary means by which socioeconomic status impacts home death.

“People with a lower socioeconomic status can’t afford to pay for private health because [the home care services organization] cannot provide all the support that’s required”… “I have met people who have higher…assets, and they’re using those assets to pay for private care, and it makes it possible for them to stay in home because they can, essentially, purchase all of the care that they need that they would get in the palliative care unit.” (Participant 1)

Finally, participants perceived health care providers as more likely to take on the responsibility for advocacy when caring for lower SES populations, noting that some clinicians go “above and beyond their duties” to get patients what they need. One explanation provided as to why clinicians were more likely to take on this responsibility for lower SES patients was recognition of the significant difference advocacy could make to patient outcomes in this population.

“In some cases if [patients] have lower socioeconomic statuses and you have people in the health care field that will advocate for you-…It’s almost like you may be empathized with a little bit more because [you] have less” (Participant 5)

### Strategies to decrease disparities

Participants identified increasing access to home-based support services, including home care support, personal support workers or other specialized care, as a strategy that might address SES-based disparities in patients’ abilities to die at home. As a part of this support, participants felt that it would be beneficial to have a designated provider for assessing, advocating and coordinating services for lower SES patients, instead of considering this to be an extra task, above and beyond one’s current duties.

“Maybe even something simple, like making it part of someone’s job to do those things, to look at how much money do [the patients] have? How can the system benefit [the patient] the most and advocate for them. But not make it part of [the provider’s] job so that they’re going above and beyond…. But make it part of someone’s job to do that, would probably be the best way [to address discrepancies]” (Participant 5)

Participants also identified enhancing support for caregivers as a strategy to improve a patient’s chances of dying at home. Providing income support for necessities like food and housing was hypothesized to relieve financial and time burden, thus providing caregivers more flexibility to engage as primary caregivers at end of life.

“Somehow providing support for their housing, for food, for being off work, and then more directly, patient care, providing more support for those people who can’t afford the extra help, but do need extra help” (Participant 4)

## Discussion

The aim of this study was to identify the key factors perceived to influence likelihood of home death, and describe the mechanisms by which they may interact with SES. Physicians in our study identified multiple factors that were perceived to influence the likelihood of home death. These factors emerged within three main categories: patient characteristics, physical environment, and support networks. Participants indicated that stability within each category was necessary for home death, and that strengths in one could supplement weaknesses in another. For example, a patient’s ability to advocate for themselves can bolster their support system by maximizing publicly funded home support. Conversely, some deficits were too large to be compensated for by strength in other categories; for example, if a patient characteristic, such as anxiety, decreases the desire to stay at home, neither an optimal physical environment, nor a robust support system would compensate for this.

Our findings suggest that, while SES is not a primary factor in determining likelihood for home death, it may influence the other main categories of determinants. Higher SES may strengthen support networks, contribute to a more stable home environment, and increase patient comfort with home death. This finding may partially explain why some studies evaluating SES on a population level did not find SES to be a significant predictor of home death [11, 25–31]; our findings suggest that the interaction between SES and other determinants such as social support or preference for place of death is complex, and thus may be obscured in population-level studies. That SES was not identified as an independent key factor of home death in our study may also result from its examination within a home-palliative care setting. In a 2015 systematic review of patients receiving specialized home care, Chen found that receiving specialist care may decrease socioeconomic inequities in access to preferred home death [39]. Similarly, Barclay found that receiving home-hospice palliative care within a continuous care model was able to eliminate disparities in the rates of transfer from home between patients classified as low or high income [40].

Preference for home death [11], and awareness of dying and realistic coping attitudes [41] have been cited as predictors of home death. Our participants indirectly linked SES to preference by way of illness understanding: Patients perceived to have less education, language barriers or lack of access to resources were believed to have poorer illness understanding, and therefore experienced greater discomfort with dying in the home. These findings are consistent with literature that has identified socioeconomic determinants that increase rates of preference for home death, including identifying as non-Hispanic white race [42], greater education level, greater income levels, greater awareness of advanced directives [43], and more supportive living arrangements [44]. These findings can be contrasted with earlier studies, which found that immigrant or direct descendants express a higher preference for home death [45] and a higher likelihood of dying at home [24]. Building on our findings, further research is needed to more completely characterize how socioeconomic factors influence preference for home death.

Finally, support systems comprised of informal caregivers and supplemented with paid-services and dedicated health care providers were felt to improve a patient’s likelihood of home death. In Canada, informal caregivers play a significant role in the provision of home care [46–48], and have been found to be predictors of home death [11, 12, 14, 27]. De Conno (1996) found that family support was a greater predictor of home death than either financial or housing conditions [27], reflecting the value of caregiver support described by our participants. Our findings further characterize the link between SES and support networks: higher SES may improve an informal caregiver’s capacity to support the patient by decreasing financial pressures, thus allowing more time for caregiving, and by compensating for gaps in informal caregiver support through greater access to paid supports. This is supported by literature. In Canada, 43% of caregivers indicated that caregiving duties disrupted their normal work routine [49]. Studies have also shown social support networks in lower SES families to have access to fewer resources, thus limiting informal caregiver capacity to provide adequate care [50, 51]. Meanwhile, caregivers with more job flexibility and disposable income are able to provide more care themselves and require fewer home care services [16]. These same caregivers may be better able to advocate for needed services [17, 20]. Finally, the economic burden that families incur while supporting home death, even within publicly funded health systems, can be significant [11, 21, 52, 53]. As Rossi et al. note in their Italian survey of family and caregivers of patients dying of cancer, a large proportion of families exhaust almost all of their savings in providing care at the end of life [54], suggesting that families with greater financial resources would face fewer barriers to home death.

### Areas of potential intervention

Strengthening support networks to facilitate home death was a primary focus of our participants, which is supported by the literature. Barclay et al. demonstrated that providing more intensive, continuous home palliative care decreased socioeconomic disparities in incidence of home death [40]. Likewise, Yamagishi et al. (2012) proposed that improving 24-h support at home is required to make home death more accessible [55].

Participants noted that a focus on caregivers, through emotional support, education, and financial assistance could also improve support network robustness, thereby contributing to improved patient state of mind. This complements the findings of Milberg et al. (2014), who found that patients who felt less supported by family had higher degrees of stress, anxiety, and increased worries about personal and financial security [56].

Better education of patients and caregivers, both around illness understanding as well as the structure of the medical system, is also a potential area of intervention. There have been documented difficulties with health literacy and physician communication in lower SES populations [57], so these populations should be especially prioritized.

### Limitations

We recognize that physicians’ perspectives may not capture the everyday realities of patients and their families at the end of life. Indeed, physicians may perceive their low SES patients as being more dependent, less responsible and less rational than higher SES patients [58], which may manifest as lack of empathy, compassion and respect for patients resulting in poor utilization of medical services [59]. Monnickendam et al. (2007) also found that social consciousness of physicians was low, and any helping behaviours were largely detached from systemic poverty-related issues [60]. As well, our findings may not be representative of the experiences of health care providers from different disciplines and settings [18, 61, 62]. While our physician participants largely appeared to demonstrate both empathic responses to patients of lower SES, and cogent analyses of the structural impacts of poverty and the systems-level policies that may improve a patient’s agency over place of death, it is important to keep in mind potential biases they may bring to this discourse.

Furthermore, we relied on a physician’s perception of their patients’ socioeconomic statuses, the accuracy of which was not validated. Indeed, participants perceived a wide variation in the socioeconomic make-up of their practices, ranging from 7.5–90%. This may be partly explained by the wide geographic disparities within the city of Toronto, but may also indicate physicians’ inaccurate perceptions of the socioeconomic status of their patients. Given that socioeconomic status was perceived to impact place of death, this finding illustrates potential benefit of implementing a formalized poverty screening tool, such as the one described by Brcic et al. [63].The physicians who participated in this study provide care to patients in their homes, which may have informed their perception of their patients’ SES. Previous studies have documented that visiting patients in their home can provide additional insight into the gaps patients may face in their care [64].

The generalizability of our findings may be limited due to the fact that our study was based within a publically funded health care system where patients have access to home care and specialized palliative care services, regardless of socioeconomic status. Results may not be as applicable, therefore, in jurisdictions where primary health care is not universally available.

### Future research

While our exploratory data suggest relationships and dependencies among the broad categories that we identified, further research with a larger and more diverse group of informants is necessary to generate a clearer model of home death.

Our respondents did not comment at length on the factors that shape preference for place of death. They did, however, note that emotional stressors do have a significant impact on patient and family comfort with home death. Further study of the determinants of patient preference would assist in addressing any modifiable factors that may arise from socioeconomic disparity.

## Conclusions

Our study provides preliminary insights into the key factors that influence home death, and defines the mechanisms by which they and socioeconomic status interact. These findings were informed by the perceptions of home palliative care physicians in an urban setting in Toronto, Canada. Our participants noted three categories of factors that affect a successful home death: patient characteristics, physical environment, and support networks. While socioeconomic status was not seen by physicians as the primary determinant of a patient’s ability to die a home, a higher SES was perceived to interact with these three categories of factors by strengthening support networks, optimizing physical home environments, and increasing patient comfort with home death. Possible areas of intervention to increase access to home death focused on better support for patients and families through increased resources and advocacy. Increasing the agency of those who prefer home death in an equitable way is a public policy imperative and should be prioritized by clinicians and policy-makers.

## Abbreviations

FTE: Full time equivalent
SES: Socio-economic status
